# Genome assembly of the Australian black tiger shrimp (*Penaeus monodon*) reveals a novel fragmented IHHNV EVE sequence

**DOI:** 10.1093/g3journal/jkac034

**Published:** 2022-02-10

**Authors:** Roger Huerlimann, Jeff A Cowley, Nicholas M Wade, Yinan Wang, Naga Kasinadhuni, Chon-Kit Kenneth Chan, Jafar S Jabbari, Kirby Siemering, Lavinia Gordon, Matthew Tinning, Juan D Montenegro, Gregory E Maes, Melony J Sellars, Greg J Coman, Sean McWilliam, Kyall R Zenger, Mehar S Khatkar, Herman W Raadsma, Dallas Donovan, Gopala Krishna, Dean R Jerry

**Affiliations:** 1 ARC Industrial Transformation Research Hub for Advanced Prawn Breeding, James Cook University, Townsville, QLD 4811, Australia; 2 Centre for Sustainable Tropical Fisheries and Aquaculture, College of Science and Engineering, James Cook University, Townsville, QLD 4811, Australia; 3 Centre for Tropical Bioinformatics and Molecular Biology, James Cook University, Townsville, QLD 4811, Australia; 4 CSIRO Agriculture and Food, St Lucia, QLD 4067, Australia; 5 Australian Genome Research Facility Ltd, Level 13, Victorian Comprehensive Cancer Centre, Melbourne, VIC 3000, Australia; 6 Laboratory of Biodiversity and Evolutionary Genomics, Biogenomics-consultancy, KU Leuven, Leuven 3000, Belgium; 7 Center for Human Genetics, UZ Leuven- Genomics Core, KU Leuven, Leuven 3000, Belgium; 8 CSIRO Agriculture and Food, Bribie Island Research Centre, Woorim, QLD 4507, Australia; 9 Faculty of Science, Sydney School of Veterinary Science, The University of Sydney, Camden, NSW 2570, Australia; 10 Seafarms Group Ltd, Darwin, NT 0800, Australia

**Keywords:** *Penaeus monodon*, Australia, genome assembly, PacBio, IHHNV EVE

## Abstract

Shrimp are a valuable aquaculture species globally; however, disease remains a major hindrance to shrimp aquaculture sustainability and growth. Mechanisms mediated by endogenous viral elements have been proposed as a means by which shrimp that encounter a new virus start to accommodate rather than succumb to infection over time. However, evidence on the nature of such endogenous viral elements and how they mediate viral accommodation is limited. More extensive genomic data on Penaeid shrimp from different geographical locations should assist in exposing the diversity of endogenous viral elements. In this context, reported here is a PacBio Sequel-based draft genome assembly of an Australian black tiger shrimp (*Penaeus monodon*) inbred for 1 generation. The 1.89 Gbp draft genome is comprised of 31,922 scaffolds (N50: 496,398 bp) covering 85.9% of the projected genome size. The genome repeat content (61.8% with 30% representing simple sequence repeats) is almost the highest identified for any species. The functional annotation identified 35,517 gene models, of which 25,809 were protein-coding and 17,158 were annotated using interproscan. Scaffold scanning for specific endogenous viral elements identified an element comprised of a 9,045-bp stretch of repeated, inverted, and jumbled genome fragments of infectious hypodermal and hematopoietic necrosis virus bounded by a repeated 591/590 bp host sequence. As only near complete linear ∼4 kb infectious hypodermal and hematopoietic necrosis virus genomes have been found integrated in the genome of *P. monodon* previously, its discovery has implications regarding the validity of PCR tests designed to specifically detect such linear endogenous viral element types. The existence of joined inverted infectious hypodermal and hematopoietic necrosis virus genome fragments also provides a means by which hairpin double-stranded RNA could be expressed and processed by the shrimp RNA interference machinery.

## Introduction

Shrimp aquaculture plays a central role in producing high-quality protein for human consumption, with global aquaculture production of the 2 major species, *Penaeus vannamei* and *P. monodon*, reaching close to 6 million tons in 2018 (FAO 2020). However, diseases, such as those caused by highly pathogenic viruses, are currently a major contributor to unfulfilled production potential (FAO 2020). Therefore, a more advanced understanding of the host defense mechanisms that suppress infection will be critical to finding solutions to viral diseases (Hauton 2017; Kulkarni *et al.* 2021; Yang *et al.* 2021). 

Initially described in insects, the viral accommodation mechanism has been hypothesized to explain why farmed shrimp highly susceptible to morbidity and mortality proceeding their initial encounter with a new virus tend to become less susceptible over time (Flegel 2020). Viral accommodation is mediated through host-genome integrated endogenous viral elements (EVEs) that can be inherited after integration into the germ line. The expressed EVE-specific double-stranded RNA (dsRNA) is then processed by the host RNA interference (RNAi) pathway, suppressing viral RNA expression levels and therefore infection loads. In the case of RNA viruses, a linear copy viral DNA (cvDNA), or circular cvDNA can be reverse transcribed by the host (Taengchaiyaphum *et al*., 2021). These DNA copies of virus RNA can then either autonomously insert into the host genome to become an EVE, or be used directly as a template for dsRNA transcription as an initial step to RNAi-mediated suppression of virus infection (Taengchaiyaphum *et al*., 2021).

Of the >50,000 known crustacean species, high-quality genome assemblies are only available for a select few taxa, driven primarily by the commercial or unique biological significance of certain species. Genome assemblies provide a reference base for functional transcriptomic studies (Yue and Wang 2017; Chandhini and Rejish Kumar 2019), aid in the positioning of genetic markers used for selective breeding (Zenger *et al.*, 2018; Houston *et al.* 2020) and provide an important resource for the examination and characterization of genomic regions of commercial or biological interest (Hollenbeck and Johnston 2018; Guppy *et al.* 2020). However, crustacean genomes have also proved immensely challenging to assemble due to their large (>2 Gbp), highly repetitive (>50%), and highly heterozygous genomes (Yuan *et al.* 2021a). To some extent, these difficulties have been alleviated by the advent of single-molecule long-read sequencing and improved genome assemblers. Extracting intact high-quality genomic DNA from muscle tissue of crustaceans like shrimp has also proved problematic and exacerbated difficulties in obtaining high-quality data from various NGS platforms (Angthong *et al.* 2020). Despite these challenges, genome assemblies highly fragmented into more than a million contigs have been reported for the penaeid shrimp species *P. vannamei* (Yu *et al.* 2015), *P. japonicus* (Yuan *et al.* 2018), and *P. monodon* (Yuan *et al.* 2018; Van Quyen *et al.* 2020). Through applying long-read sequencing and HiC scaffolding, less fragmented high-quality genomes have also been achieved recently for *P. vannamei* (Zhang *et al.* 2019), *P. monodon* (pseudo-chromosome level) (Uengwetwanit *et al*., 2021), and *P. japonicus* (Kawato *et al.* 2021).

Reported here is a high-quality draft genome assembly of a single-generation inbred male *P. monodon* from eastern Australia, a population genetically distinct from others across their South East Asian, Indo-Pacific, and East African distribution (Vu *et al.* 2021). We report and resolve the genomic structure of an EVE of infectious hypodermal and hematopoietic necrosis virus (IHHNV) comprised of repeated, inverted, and jumbled IHHNV genome fragments. We discuss the disease detection implications of false PCR-positives for infectious IHHNV, and how the EVE might have originated.

## Materials and methods

### Shrimp breeding and selection for sequencing

A second-generation (G2) male *P.* *monodon* that had undergone a single cycle of inbreeding was selected for genomic sequencing. The original wild-caught broodstock were collected from a Queensland east coast location (approximately 17.3°S, 146.0°E) in September 2013. In October 2013, 14 first-generation (G1) families were produced from the brood stock at Seafarm Flying Fish Point hatchery (approximately 17.5°S, 146.1°E). In February 2015, pleopod tissue was sampled from 50 female and 50 male G1 broodstock. These tissues were genotyped [using 2 × 60 SNP panels (Sellars *et al.* 2014)] to identify the parental origin of each broodstock and to select related mating pairs to generate the inbred G2 progeny. In August 2015, groups of 50 juvenile males from 5 inbred G2 families were euthanized to collect muscle tissue from the first abdominal segment for sequencing and the second most anterior pair of pleopods for genotyping. These tissues, as well as the remainder of each shrimp (archived source of tissue for sequencing) were snap frozen under dry ice pellets and stored at −80°C. Each shrimp was then genotyping using the 120-SNP panel (Sellars *et al.* 2014) and a genome-wide SNP assay based on DArTSeq (Guppy *et al.* 2020). After ranking the 50 males based on inbreeding coefficient (F) and multilocus heterozygosity (MLH) data from the 120-SNP panel, the individual (named Nigel) with the highest inbreeding coefficient was chosen for genomic sequencing. The choice was confirmed using a genome-wide SNP assay based on DArTSeq of the top 5 inbred shrimp based on the 120-SNP panel which recovered the same ranking (Nigel: MLH of 0.231 and F of 0.271).

### DNA extraction, library preparation, and genome sequencing

Multiple extraction methods were trialed to generate intact high-quality genomic DNA from stored muscle tissue of the single selected inbred shrimp. All DNA extractions and sequencing runs were carried out at the Australian Genome Research Facility (AGRF), Melbourne, Australia. For Illumina sequencing, the MagAttract HMW DNA kit (QIAGEN) was used and PCR-free fragment shotgun libraries were prepared using the “with-bead pond library” construction protocol described by Fisher *et al.* (2011) with some modifications (Supplementary Material 1). The library was sequenced on 2 HiSeq 2500 lanes using a 250 bp PE Rapid sequencing kit (Illumina). The same DNA was also used to create a 10× Genomics Chromium library as per the manufacturer’s instructions, which was sequenced on 2 HiSeq 2500 lanes using a 250 bp PE Rapid sequencing kit. For PacBio sequencing, the following DNA extraction methods were used with varying success: MagAttract HMW DNA kit (QIAGEN), Nanobind HMW Tissue DNA kit-alpha (Circulomics), and CTAB/Phenol/Chloroform (Supplementary Table 1). Libraries were prepared using the SMRTbell Template Prep Kit 1.0 (PacBio), loaded using either magbeads or diffusion, and sequenced using the Sequel Sequencing Kits versions 2.1 and 3.0 on a PacBio Sequel (Supplementary Table 1). The same muscle tissue was also used to prepare 3 Dovetail Hi-C libraries according to the manufacturer’s instructions. Two libraries were sequenced on a shared lane of a NovaSeq S1 flow cell, and a third library was sequenced on 1 lane of a NovaSeq SP flow cell, with both sequencing runs generating 100 bp paired-end reads.

### Genome assembly

The quality of the initial short-read genome assemblies using either DISCOVAR de novo (Weisenfeld *et al.* 2014) with Illumina data, or Supernova (Weisenfeld *et al.* 2017) with 10× Genomics Chromium data were poor. The most contiguous assembly was achieved using wtdbg2/redbean (Version 2.4; Ruan and Li 2019) with 75× times coverage of PacBio data, setting the estimated genome size to 2.2 Gb, but without using the wtdbg2 inbuilt polishing. The raw assembly was subjected to 2 rounds of polishing using the PacBio subreads data in arrow (Version 2.3.3, github.com/PacificBiosciences/GenomicConsensus) and 1 round of polishing using the Illumina short-read data in pilon (Version 1.23, Walker *et al.* 2014). Scaffolds were constructed in 2 steps. Medium-range scaffolding carried out using 10× Genomics Chromium data with longranger (Version 2.2.2, https://support.10xgenomics.com/genome-exome/software/downloads/latest) and ARCS (Version 1.0.6, Yeo *et al.* 2017), while long-range scaffolding was performed using dovetail Hi-C data, and intra- and interchromosomal contact maps were built using HiC-Pro (Version 2.11.1, Servant *et al.* 2015) and SALSA (commit version 974589f, Ghurye *et al.* 2017). This genome assembly was then submitted to NCBI GenBank, which required the removal of 2 small scaffolds and the splitting of 1 scaffold. The overall quality of the final V1.0 genome was assessed using BUSCO, through mapping of RNA-seq and Illumina short-reads using HiSAT2 (version 2.1.0, Kim *et al.* 2019) and Merqury (Rhie *et al.* 2020).

### Repeat annotation

Repeat content was assessed with de novo searches using RepeatModeler (V2.0.1) and RepeatMasker (V4.1.0) via Dfam TE-Tools (V1.1, https://github.com/Dfam-consortium/TETools) within Singularity (V2.5.2, Kurtzer *et al.* 2017). Additionally, tandem repeat content was determined using Tandem Repeat Finder (V4.0.9, Benson 1999) within RepeatModeler. Analyses and plotting of interspersed repeats were carried out as per Cooke *et al*. (2020), github.com/iracooke/atenuis_wgs_pub/blob/master/09_repeats.md). Additionally, the genomes of the Black tiger shrimp (Thai origin, www.biotec.or.th/pmonodon; Kim *et al.* 2019), Whiteleg shrimp (*P. vannamei*, NCBI accession: QCYY00000000.1; Zhang *et al.* 2019), Japanese blue crab (*Portunus trituberculatus*, gigadb.org/dataset/100678; Tang *et al.* 2020), and Chinese mitten crab (*Eriocheir japonica sinensis*, NCBI accession number: LQIF00000000.1) were run through the same analyses for comparison.

### Gene prediction and annotation

In order to generate an RNA-seq-based transcriptome, raw data from a previous study (NCBI project PRJNA421400; Huerlimann *et al.* 2018) were mapped to the masked genome using STAR (Version 2.7.2b; Dobin *et al.* 2013), followed by Stringtie (Version 2.0.6; Pertea *et al.* 2015) (Supplementary Table 2). Additionally, the IsoSeq2 pipeline (PacBio) was used to process the ISO-seq data generated in this study (Supplementary Table 2). Finally, the genome annotation was carried out in MAKER2 (v2.31.10; Cantarel *et al.* 2008; Holt and Yandell 2011; Campbell *et al.* 2014) using the assembled RNA-seq and ISO-seq transcriptomes together with protein sequences of other arthropod species (Supplementary Table 3).

### EVE analysis

BLASTn using a 3,832 bp IHHNV EVE Type A sequence detected in Australian *P. monodon* (Au2005; EU675312.1) as a query identified a potential EVE in Scaffold_97 of the *P. monodon* genome assembly. The EVE was unusual in that it comprised of repeated, inverted and jumbled fragments of an EVE Type A sequence. The nature and arrangement of EVE fragments was initially determined manually and the relative sequence positions of matching fragments within the EVE and scaffold sequence was determined using QIAGEN CLC Genomics Workbench 18.0 (https://digitalinsights.qiagen.com/). To confirm the authenticity of the Scaffold_97 EVE (S97-EVE), 6 PCR primer sets were designed using Primer 3 v.0.4.0 (Koressaar and Remm 2007; Untergasser *et al.* 2012) to amplify each EVE boundary and 2 internal sequences (Supplementary Table 4). DNA was extracted from ∼10 mg gill tissue stored at −80°C from the *P. monodon* sequenced using DNAeasy kit spin columns (QIAGEN). DNA was eluted in 50 µl EB buffer, aliquots were checked to DNA concentration and purity using a Nanodrop 8000 UV spectrophotometer and the remainder was stored at −20°C. As DNA yields were low (9–38 ng/µl), a 1.0-µl aliquot of each sample was amplified in 10 µl reactions incubated at 30°C for 16 h as described in the REPLI-g Mini Kit (QIAGEN).

Each PCR (25 µl) contained 2 µl REPLI-g amplified gill DNA, 1× MyTaq Red Mix (Bioline), 10 pmoles each primer and 0.25 µl (1.25 U) MyTaq DNA Polymerase (Bioline). Thermal cycling conditions were 95°C for 1 min followed by a 5-cycle touch-down (95°C for 30 s, 60°C to 56°C for 30 s, 72°C for 20 s), 30 cycles of the same using an anneal of 55°C for 30 s, followed by 72°C for 7 min and a 20°C hold. For seminested PCR using the 1b and 4b primer sets, 1 µl each PCR (either neat or diluted 1:5 to 1:10 depending on PCR product amount) was amplified similarly for 30 cycles using an anneal step of 55°C for 30 s. Aliquots (5–10 µl) of each reaction were electrophoresed in a 1.0% agarose-TAE gel containing 0.1 µl/ml ethidium bromide, and a gel image was captured using a Gel Doc 2000 UV transilluminator (Bio-Rad). Each amplicon was purified using a spin column (QIAGEN) and sequenced at the AGRF, Brisbane. The quality of sequence chromatograms was evaluated and consensus sequences for each amplicon were generated using Sequencher 4.9 (Gene Codes Corp.).

## Results and discussion

### DNA extraction, library preparation, and genome sequencing

In total, 158 Gb (72× coverage) of Illumina, 494 Gb (224× coverage) of 10× Genomics Chromium, 165 Gb (75× coverage) of PacBio Sequel, and 119 Gb (54× coverage) of DoveTail data were generated (Table 1). While the MagAttract HMW DNA kit (QIAGEN) was suitable for Illumina sequencing (PCR-free shotgun libraries and 10× Genomics Chromium), using this DNA resulted in poor PacBio Sequel sequencing runs (Supplementary Table 1). Runs consistently showed low yield and short-fragment lengths, despite relatively high molecular weight DNA. However, DNA extracted with the Nanobind HMW Tissue DNA kit-alpha (Circulomics, Inc., Baltimore, USA) showed better sequencing performance (higher yield and fragment length; Supplementary Table 1). Furthermore, diffusion loading of the PB Sequel resulted in better results than magbead loading. DNA derived from either extraction method was unsuitable for Oxford Nanopore Technology (ONT) sequencing due to it rapidly blocking the pores (data not shown).

**Table 1.** Illumina, PacBio, 10× Genomics, and DoveTail sequencing data used for the assembly and scaffolding of the black tiger shrimp genome.

Sequencing platform	Paired end reads	Yield (Gb)	Coverage	GenBank accessions
Illumina (250 bp PE)	315 M	158	72×	SRR10713996, SRR10713997
PacBio Sequel	N/A	165	75×	SRR10713990–SRR10713995
				SRR10713998–SRR10714025
10× Genomics (250 bp PE)	987 M	494	224×	N/A
DoveTail (100 bp PE)	1.2 B	119	54×	N/A

Sequence quality issues associated with DNA extraction have also been noted in other shrimp genome assembly reports (Zhang *et al.* 2019; Uengwetwanit *et al*., 2021). The patterns seen in the PacBio sequencing results (short polymerase read lengths despite high quality libraries), coupled with the inability to successfully sequence *P. monod*on using ONT technology (immediate pore blockage), can be explained by high amounts of polysaccharides and polyphenolic proteins co-extracting with the DNA. This has also been mentioned by Angthong *et al.* (2020), who also present an alternative DNA extraction method to the Circulomics Nanobind HWM Tissue DNA extraction kit suggested here.

### Genome assembly and quality assessment

As reported by other Penaeid shrimp genome sequencing projects (Zhang *et al.* 2019; Uengwetwanit *et al*., 2021; Yuan *et al.* 2021a), sequencing and assembly of the Australian *P. monodon* genome proved problematic due to its large size, substantial heterozygosity, and prevalence of repeat elements. The de novo assembly of the PacBio data resulted in 47,607 contigs (contig N50: 77,900 bp) a total of 1.90 Gbp in size (Table 2). After medium-range scaffolding with 10× Genomic Chromium data and long-range scaffolding with Dovetail sequences, the resulting scaffolded assembly contained 1.89 Gbp across 31,922 scaffolds (scaffold N50: 496,398 bp; Table 2). Assuming a genome size of 2.2 Gbp (Huang *et al.* 2011), this scaffolded assembly covers 85.9% of the projected *P. monodon* genome (Table 2). This is slightly lower than the 90.3% recently achieved for the same species in Thailand (Uengwetwanit *et al*., 2021), and higher than the 67.7% achieved for *P. vannamei* (Zhang *et al.* 2019), which has a slightly larger genome. Altogether, 98.1% of the Illumina DNA short-read data mapped to the raw assembly, and Merqury showed a k-mer completeness of 76.9%, a quality value of 24.7 and an error rate of 0.0033. BUSCO (V3; Simão *et al.* 2015), using the Arthropoda odb9 database (Zdobnov *et al.* 2017), estimated the Australian *P. monodon* genome assembly to be 86.8% complete (gene *n* = 1,066; 85.8% single copy; 1.0% duplicated; 4.5% fragmented; 8.7% missing; Table 2). These assembly metrics are comparable to those achieved for the Thai *P. monodon* assembly (C 87.9%, S 84.8%, D 3.1%, F 4.0%, M 8.0%; Uengwetwanit *et al*., 2021) and slightly better than those achieved for the *P. vannamei* assembly (C 78.0%, S 74.0%, D 4.0%, F 4.0%, M 18.0%; Zhang *et al.* 2019), both analyzed with the same database and BUSCO version (Table 2).

**Table 2.** Summary of assembly statistics for the Australian and Thai *P. monodon*, and *P. vannamei* genomes.

Metrics	*P. monodon* (Australia)	*P. monodon* (Thailand)	*P. vannamei*
No. of contigs	47,607	70,380	50,304
Largest contig	1,147,530	1,387,722	739,419
Total length of contigs	1.89 Gb	2.39 Gb	1.62 Gb
Contig N50	78 kb	79 kb	58 kb
No. of scaffolds	31,922	44	–
Largest scaffold	21.70 Mb	65.87 Mb	–
Total length of scaffolds	1.89 Gb	1.99 Gb	1.66 Gb
Scaffold N50	0.50 Mb	49.0 Mb	0.60 Mb
Projected genome size	2.20 Gb	2.20 Gb	2.45 Gb
Percentage covered by scaffolds	86.1%	90.3%	67.7%
GC (%)	35.6	36.6	35.7
Complete BUSCOs (C)	86.8	87.9	78.0
Complete and single-copy BUSCOs (S)	85.8	84.8	74.0
Complete and duplicated BUSCOs (D)	1.0	3.1	4.0
Fragmented BUSCOs (F)	4.5	4.0	4.0
Missing BUSCOs (M)	8.7	8.0	18.0
No. of predicted gene models	35,517	31,640	25,596
No. of protein-coding genes	25,809	30,038	–
No. of genes annotated in interproscan	17,158	20,615	–
References	This study	Uengwetwanit *et al.* (2021)	Zhang *et al.* (2019)

### Functional and repeat annotation

**Fig. 1.**
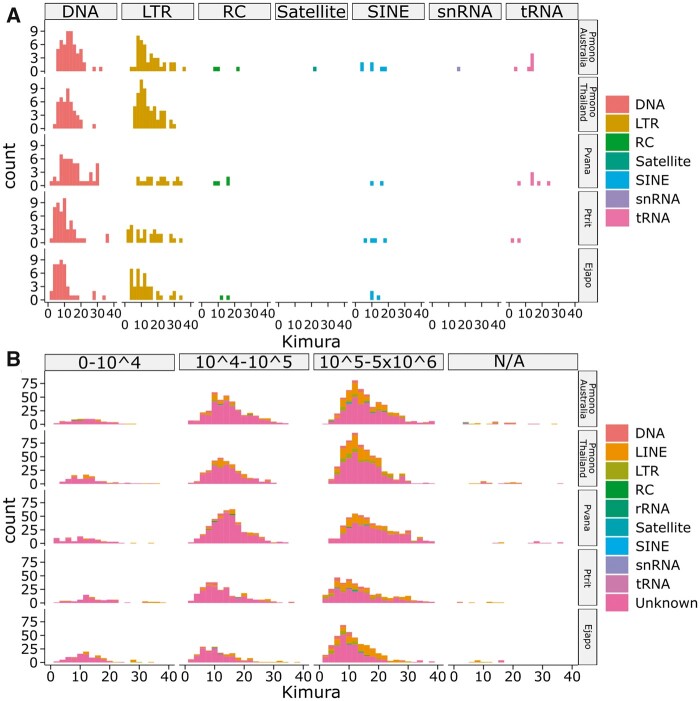
Kimura distances of repetitive sequences in the genome assemblies of Australian black tiger shrimp (Pmono Australia, *P. monodon*, NCBI accession: JAAFYK000000000, this study) Thai black tiger shrimp (Pmono Thailand, *P. monodon*, Pmono Thailand, Uengwetwanit *et al*. 2021), Whiteleg shrimp (Pvana, *Penaeus vannamei*, NCBI accession: QCYY00000000.1, Zhang *et al.* 2019), Japanese blue crab (Ptrit, *Portunus trituberculatus*, gigadb.org/dataset/100678, Tang *et al.* 2020), and Chinese mitten crab (Ejapo*, Eriocheir japonica sinensis*, NCBI accession: LQIF00000000.1) determined by using either (a) repeat length or (b) repeat class.

The functional annotation using RNA-seq, ISO-seq, and protein information, identified 35,517 gene models, of which 25,809 were protein-coding and 17,158 were annotated using interproscan (Table 2). Similar numbers of genes were annotated in the Thai *P. monodon* (Uengwetwanit *et al*., 2021) and *P. vannamei* (Zhang *et al.* 2019) assemblies. Repeat content in the Australian *P. monodon* assembly (61.8%) was high, like in the Thai *P. monodon* assembly (62.5%; Uengwetwanit *et al*., 2021), and substantially higher than in genome assemblies of *P. vannamei* (51.7%; Zhang *et al.* 2019), *P.* *trituberculatus* (45.9%, Tang *et al.* 2020) or *E.* *japonica sinensis* (35.5%, LQIF00000000.1) (Supplementary Table 5; Fig. 1). Interestingly, simple sequence repeats (SSRs) that dominated in prevalence (30.0%) in the Australian *P. monodon* assembly were less prevalent (23.9%) in the Thai *P. monodon* assembly (Uengwetwanit *et al*., 2021), similarly prevalent (27.1%) in the *P. vannamei* assembly, but far less prevalent in the genome assemblies of either the Japanese blue (16.9%) or Chinese mitten crab (7.9%) (Supplementary Table 5; Fig. 1). Such high SSR levels have been linked to genome plasticity and adaptive evolution facilitated through transposable elements (Yuan *et al.* 2021b). In addition to SSRs, the Australian *P. monodon* assembly contained 9.8% long-interspersed nuclear elements, 2.5% low complexity repeats, 2.0% DNA transposons, 1.6% long-terminal repeats, 0.51% small-interspersed nuclear elements, 0.1% satellites, 0.01% small RNA repeats, and 15.4% unclassified repeat element types (Supplementary Table 5; Fig. 1). Broad comparisons of the major repeat types in the genome assemblies of *P. monodon*, *P. vannamei*, *P.* *trituberculatus*, and *E. japonica sinensis* based on kimura distances showed them to be relatively conserved across all 4 crustacean species (Fig. 1). At all lengths and levels of divergence, unknown repeats dominated, with a large proportion of these >100 kb in size (Fig. 1a). Repeat patterns shared across the 4 species were further highlighted when unknown reads were removed, and repeats split into major classes (Fig. 1b).

### IHHNV-EVE rearrangement in the Australian *P. monodon* genome

**Fig. 2.**
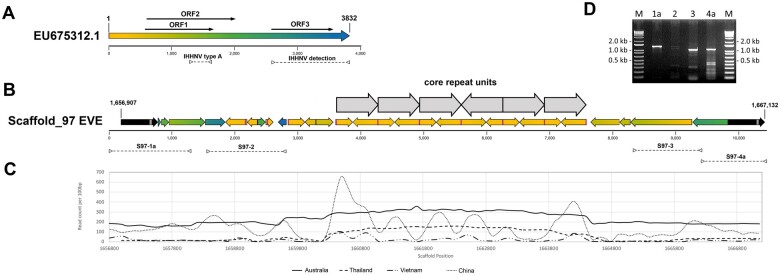
a) Schematic diagram of a 3,832 bp ssDNA genome of infectious hypodermal and hematopoietic necrosis virus (IHHNV) showing the relative positions of coding sequences (arrows) for the virus replicase (ORF1), NS1 nonstructural protein (ORF2), and viral capsid protein (ORF3). A color gradient was applied to visualize relative genome positions. b) Schematic diagram of the positions and orientations of IHHNV genome fragments comprising the Scaffold_97 EVE (S97-EVE). The orientations of the IHHNV fragments (colored arrows) and the flanking repeated 591/590 bp host sequence (black arrows) are shown by arrow directions. The origins of the S97-EVE fragments relative to their positions in a linear IHHNV-EVE (see a) are identified by color. The 10,226 bp S97-EVE resided between positions 1,656,907 and 1,667,132 in the 2,608,951 bp Scaffold_97 sequence. The larger gray arrows identify the positions and orientations of at least 6 core repeat blocks comprising of 2 smaller inverted repeats. Gray vertical bars show the location of a 34 bp sequence in each flanking repeat capable of folding into a stable secondary structure. The purple vertical bars show the locations of the 18 bp palindromic sequence present at the boundaries of each RU and partial RU. Dashed lines (>–<) identify the regions amplified by the 4 PCR tests S97-1a, S97-2, S97-3, and S97-4a. c) Coverage depth across the S97-EVE sequence of raw short reads used to assemble genome scaffolds of *P. monodon* from Australia (this study), Thailand (Uengwetwanit *et al.*, 2021), Vietnam (Van Quyen *et al.* 2020), and China (Yuan *et al.* 2018). d) Agarose gel image showing DNA products amplified by the S97-1a, S97-2, S97-3, and S97-4a PCR tests.

Sequences homologous to a 3,832 bp linear IHHNV-EVE (Au2005, type A) found to occur in some Australian *P. monodon* (Krabsetsve *et al.* 2004) were identified in Scaffold_97 (S97, 2,608,951 nt). However, rather than representing an intact linear copy of this EVE, the S97-EVE comprised a 9,045 bp stretch of jumbled, repeated, and inverted IHHNV fragments flanked by 2 repeated 591/590 bp (flanking repeat) sequences (Fig. 2). Alignments identified most fragments to be jumbled relative to their location in the Au2005 IHHNV-EVE sequence, and the expanded EVE length to be due to replicated short sequences originating from 5′-terminal genome regions. Fragments positioned at the S97-EVE extremities generally originated from the central and downstream regions of the Au2005 IHHNV-EVE sequence and were consistently orientated inwards. The central S97-EVE region comprised a block of at least six 661 bp repeat units (RUs). Each RU was comprised of 2 inward-facing sequences either (A) 398 bp or (B) 263 bp in length that mapped to the same region (94–501 and 94–368, respectively) at the 5′-terminus of the Au2005 IHHNV-EVE (Fig. 2b, gray arrows). In total, 83% of the Au2005 IHHNV-EVE sequence was identified to be covered by genome fragments present in the S97-EVE, with those present being on average 99.3% identical.

The inverted A and B sequences comprising each RU contain RNA transcription regulatory signals of the IHHNV P2 promoter (Shike *et al.* 2000; Dhar *et al.* 2007, 2010, 2011). Both initiated at a sequence (5′-GTCATAGGT…) mapping precisely to a G nucleotide residing immediately downstream of the inversion point (|) of an 18 bp inverted repeat (5′-.TTACAACCTATGAC|GTCATAGGTCCTATATAAGAGT.-3′) located 2 bp upstream of the TATA-box element (5′-TATATAA-3′) of the P2 transcriptional promoter (Dhar *et al.* 2007, 2010, 2011). The A and B repeat components in each RU of the 6 blocks were orientated 5′|B-A|B-A|B-A|A-B|B-A|B-A|3′, with those in RU4 being reversed compared to the others. Due to the A and B repeat components being inverted, the 18 bp inverted repeat (i.e. 5′-.ACTCTTATATAGGACCTATGAC|GTCATAGGTCCTATATAAGAGT.-3′) was reconstructed at each of the 5 RU junction sites irrespective of which 2 repeat components (A|A, A|B, or B|B) were joined (Fig. 2b, purple bars). This arrangement generated a 544-bp inverted repeat (263× 2 + 18) for sequences extending from either A|B or B|B RU junctions, or a 1,902-bp inverted repeat (661×2 + 263×2 + 18×3) for the long complimentary sequence stretches extending outwards from the A|A components at the RU3|RU4 junction to the end of repeat component A of RU2 and the equivalent position of repeat component B in RU5. However, relating to the descriptions of this unusual EVE segment, it is important to note that no single long read was obtained that traversed the entire 6 RU blocks into flanking unique S97-EVE sequences (Fig. 2). Combined with short-read numbers generated using various sequencing methods being substantially elevated at positions mapping to each block RU (Fig. 2c), the likelihood of the block comprising more than 6 RUs remains to be established.

DNAFold and RNAfold analyses showed the 18 bp inverted repeat, the inverted A and B repeat components of each RU and the longer complimentary sequences that stretched through multiple RUs to all have potential to form highly stable simple to complex secondary structures as either ssDNA or ssRNA (data not shown). Discrete DNA secondary structures are known to have roles in mediating recombination in mobile genetic elements (Bikard *et al.* 2010) and in the genomes of parvoviruses like the extensively studied adeno-associated virus (AAV), structures formed by inverted terminal repeat (ITR) sequences play critical roles in initiating genomic ssDNA replication, genomes forming circular extrachromosomal dsDNA episomes and genomic integrating into host chromosomal DNA (Kotin *et al*., 1991; Cotmore and Tattersall 1996; Yang *et al.* 1997; Schnepp *et al.* 2005). The mechanisms leading to the A and B inverted repeat sequences forming the 661 bp RUs and their apparent multiplication in the central region of the S97-EVE remains unknown. However, their existence is consistent with integrated AAV proviral DNA structures being observed to contain head-to-tail tandem arrays of partial ITR sequences and for genomic rearrangements occurring via deletion and/or rearrangement-translocation at the integration site (Yang *et al.* 1997).

The 18 bp inverted repeat at the S97-EVE RU junctions also occurred at the upstream RU1 and downstream RU6 boundaries of the RU block. However, unlike those at the internal RU junctions which extended into the same downstream Au2005-EVE sequence including the TATA-box element (Krabsetsve *et al.* 2004; Dhar *et al.* 2007, 2010, 2011), the outer half of each inverted repeat flanking the RU-block extended into sequences toward the 5′ end of the IHHNV genome (Supplementary Fig. 1). Three disparate partial RU sequences (pRUa, pRUb, pRUc) associated with four 18-bp inverted repeats also resided just upstream of the 6 RU block. Like RU1 and RU6, one side of each inverted repeat possessed variable lengths of sequence extending toward the IHHNV genome 5′-terminus (Supplementary Fig. 1).

In some IHHNV strains, the sequence immediately upstream of the 18-bp inverted repeat comprises a second imperfect 39–40 bp inverted repeat. With an IHHNV strain detected in Pacific blue shrimp (*Penaeus stylirostris*) sampled from the Gulf of California in 1998 (Shike *et al.* 2000, AF273215.1), the 5′-genome terminus upstream of it consisted of an 8 bp portion of the downstream 18 bp inverted repeat (Supplementary Fig. 1). In the S97-EVE, the 18 bp inverted repeats associated with each terminal RU or upstream pRU extended 18–38 bp into the 39–40 bp inverted repeat (Supplementary Fig. 1). Of interest, with the first pRU occurring in the S97-EVE (5′-pRUa), the 93 bp sequence abutting the 18 bp inverted repeat was also identical to the 5′-terminal sequence reported for the Au2005 IHHNV-EVE found in *P. monodon* sampled from farms in Australia in 1993/1997 (Krabsetsve *et al.* 2004; EU675312.1).

To confirm that the fragmented and jumbled nature of the S97-EVE was not an assembly artifact, regions spanning each EVE extremity to unique host sequences positioned just beyond the 591/590 bp flanking repeats, as well as 2 internal regions each spanning conjoined nonrepeated EVE fragments were amplified by PCR (Supplementary Table 4; Fig. 2d). Amplicons of the expected sizes were clearly amplified by each extremity PCR test (S97-1a and S97-4a) and the S97-3 internal PCR test (Fig. 2d). The other internal PCR test (S97-2) also generated a 1,337 bp amplicon of the expected size, as well as one ∼200 bp shorter, but in relatively lower abundance. Using each extremity PCR product as template, seminested PCR tests using an alternative internal EVE-specific primer also produced amplicons of the expected shorter sizes, and their authenticity was confirmed by sequence analysis (data not shown).

### *Penaeus monodon* repeat sequences flanking the IHHNV-EVE

BLASTn and BLASTx searches did not identify any homologs of the 591/590 bp flanking repeat sequence in GenBank. However, searches of the *P. monodon* genome assembly identified long closely related sequences in hundreds of other scaffolds (data not shown). The searches also highlighted the presence of a 34-bp sequence (5′-.ATGACTCCTCCCCCATAGATAGGGGCGGAGTCAT.-3′) in each flanking repeat (Fig. 2b, gray bars, upstream repeat position 1,657,364–1,657,397; downstream repeat position 1,667,000–1,667,033) that was also present in 178 other scaffolds at >80% identity. DNAFold and RNAfold analyses showed the sequence and its reverse compliment to fold into stable hairpin structures as either ssDNA (ΔG = −10.44/−11.92, Tm = 83.8/85.7°C) or ssRNA (ΔG = −20.40/−23.70). However, whether this or other sequences in the host flanking repeat interact with IHHNV genome sequences and proteins to facilitate recombination and site-specific integration remains to be investigated. In this regard, the flanking host repeat possessed a 5′..CTTACTTACACTTG..3′ tetramer repeat, which to the 5′-side of the S97-EVE was located 33 bp upstream of the IHHNV CTTA.. sequence at the host/S97-EVE junction, much like the host tetramer repeats well characterized to be pivotal to the AAV genome integrating at a specific location in human chromosome 19 (Kotin *et al.* 1992; Linden *et al.* 1996).

### Comparison to jumbled IHHNV-EVEs in other *P. monodon* genome assemblies

BLASTn searches of the most comprehensive genome assembly of a *P. monodon* from Thailand (NSTDA_Pmon_1, GCA_015228065.1, Uengwetwanit *et al*., 2021) identified Scaffold_35 (S35) containing 2 disparate aggregations of jumbled IHHNV-EVE Type A fragments (S35-EVE1 = 7,888 bp; S35-EVE2 = 16,310 bp) each flanked by >500 bp host repeats near identical in sequence to those flanking the S97-EVE (Table 3). Compared to the S97-EVE, 2,328 bp of S35-EVE1 sequence immediately downstream of the 5′ 592 bp host repeat, except for a 166 bp deletion, and 647 bp of sequence immediately upstream of the 3′ 591 bp host repeat, were identical. Further inwards, however, the order and arrangement of EVE fragments diverged.

**Table 3.** Detection and notable features of IHHNV-EVE sequences identified in other genomes of *P. monodon*.

Reference genome IDs	Notable EVE features				
	Start	End	Length (bp)	Orientation	Homology (%)
*P. monodon* Thailand (Uengwetwanit *et al.*, 2021)					
* Scaffold 35 EVE-1*	770,236	778,124	7,888		
* *RU1	772,730	773,391	661	Minus	99.9
* *RU2	773,450	774,111	661	Plus	100.0
* *RU3	774,170	774,831	661	Minus	99.9
* * RU4	774,890	775,551	661	Plus	97.9
* Scaffold 35 EVE-2*	862,618	878,928	16,310		
* *RU1	866,534	867,145	611	Minus	79.4
* *RU2	867,204	867,791	587	Plus	81.3
* *RU3	867,840	868,467	627	Minus	83.5
* *RU4	868,515	869,130	615	Plus	80.0
* *RU5	872,127	872,754	627	Plus	78.9
* *RU6	872,799	873,434	635	Minus	90.0
* *RU7	873,492	874,152	660	Plus	97.2
* *RU8	875,469	876,168	699	Plus	92.1
*P. monodon* Vietnam (Pmod26D_v1; GCA_007890405.1)					
* *VIGR010059916.1 EVE (4,003 bp)			4,003		98.4
* *VIGR010211091.1 EVE (1,917 bp)			1,917		99.0
* *VIGR010168684.1 EVE (2,220 bp)			2,220		98.9
*P. monodon* China (Pmon_WGS_v1, GCA_002291185.1)					
* *gb|NIUS011382605.1 (645 bp)			645		98.9
* *gb|NIUS011109800.1 (848 bp)			848		98.3

As in the S97-EVE, the central region of the S35-EVE1 contained a block of 4 ×661 bp RUs each comprised of the same inward facing (A) 398 bp and (B) 263 bp repeats but ordered 5′|A-B|B-A|A-B|B-A|3′, thus making a 2,877-bp inverted repeat with an inversion point at the RU2-RU3 boundary. Also, like the 97-EVE, each S35-EVE RU was flanked by same 18 bp inverted repeat sequence, with those upstream of RU1 and downstream of RU4 extending 17–33 bp into a 41 bp imperfect inverted repeat sequence located immediately upstream toward the 5′-genome termini in some IHHNV strains (Supplementary Fig. 2). However, unlike the RU block in the S97-EVE, each of the 3 internal S35-EVE RU boundaries comprised of 2 ×18 bp inverted repeats flanking the complete 41 bp imperfect inverted repeat (Supplementary Fig. 2). This revised the RU junction to the inversion point in longer imperfect inverted repeat, rather than the inversion point of the 18 bp inverted repeat. DNAfold and RNAfold analyses showed that the 41 bp inverted repeat and its reverse compliment sequence could fold into stable hairpin structures as either ssDNA (ΔG = −14.18/−14.86, Tm = 73.6/75.6°C) or ssRNA (ΔG = −22.50/−25.00).

The larger S35-EVE2 sequence differed in the arrangement and homology of up to 8 RUs, possibly composed of 2 entirely duplicated inward-facing EVE fragments (Table 3). The IHHNV-EVE fragments in S35-EVE1 contained 72% of the Au2005 IHHNV-EVE sequence with 98.8% homology, on average. In contrast, IHHNV-EVE fragments in S35-EVE2 region only contained 53% of the IHHNV-EVE sequence with 97.5% homology, on average.

BLASTn searches of the genome assembly of a *P. monodon* from Vietnam (Pmod26D_v1, GCA_007890405.1, Van Quyen *et al.* 2020), using the 9,045 bp S97-EVE and 3,832 bp linear Au2005 Type A IHHNV-EVE sequences identified 3 short contigs (*VIGR010059916.1*, 4,003 nt; *VIGR010168684.1*, 2,220 nt; *VIGR010211091.1*, 1,917 bp) also comprised of jumbled IHHNV-EVE Type A-like fragments (Table 3). In 2 of the contigs, the stretches of jumbled EVE fragments neighbored either a complete (590 bp) or incomplete (356 bp) host repeat sequences like those flanking the S97-EVE. BLASTn searches of a genome assembly of a *P. monodon* from Shenzhen, China (Pmon_WGS_v1, GCA_002291185.1) also identified evidence of an EVE comprised of jumbled IHHNV genome fragments (Table 3), and despite contig lengths being short, it was also being flanked by the same repeated host sequence flanking the S97-EVE (data not shown). While more complete higher quality genome assemblies would add confidence, the insertion locations of the jumbled EVEs present in the genome assemblies of the *P. monodon* from Vietnam and China appear shared with those the Australian S97-EVE and Thai S35-EVE1, with the second less-related jumbled S35-EVE2 in the Thai genome residing at a nearby site. Interestingly, BLASTn searches of the genome assemblies of *P. monodon* from Australia, Thailand, Vietnam, or China identified no evidence of linear IHHNV-EVE forms.

### Origins and implications of jumbled IHHNV-EVEs

While varying in lengths, the amalgamations of reordered, inverted, and repeated IHHNV genome fragments comprising the EVEs detected in Scaffold_97 (S97) of the Australian *P. monodon* assembly (this study) and in Scaffold_35 (S35) of the Thai *P. monodon* assembly (Uengwetwanit *et al.*, 2021) share an integration site as well as structural and sequence similarities with the partial EVE sequences detected in short contigs of genome assemblies of *P. monodon* originating from Vietnam and China (as outlined above). These similarities are suggestive of a progenitor IHHNV genome becoming stably integrated as an EVE prior to *P. monodon* becoming dispersed widely across its current distribution range. Such an ancient event would also support differences noted, for example, in EVE fragment composition, central RU numbers, and the nature of the conserved inverted-repeat sequences defining the boundaries of the RUs. Furthermore, the conservation of the inverted-repeat sequences at the RU boundaries and their potential to form stable ssDNA folding structures suggests a potential role in their apparent multiplication.

The IHHNV P2 RNA transcriptional promoter motifs, including the 18 bp inverted repeat sequences and TATA-box (Shike *et al.* 2000; Dhar *et al.* 2014; Silva *et al.* 2014), at the RU boundaries have potential to facilitate transcription of various virus-specific sense and antisense ssRNA sequences. RNA transcribed from them would then be capable of forming long virus-specific dsRNA or hairpin dsRNAs, potentially in high abundance due to their repeated nature. If so, such virus-specific antisense RNAs or dsRNA forms processed through the RNAi machinery of *P. monodon* (Su *et al.* 2008; Attasart *et al.* 2010, 2011; Dhar *et al.* 2014) could provide resilience against IHHNV infections progressing to become acute and cause disease. Such an advantage might promote the selection of *P. monodon* carrying this form of IHHNV-EVE, particularly in circumstances when shrimp are specifically selected or bred for aquaculture robustness. Selection for the EVE over several years would also be consistent with the viral accommodation model hypothesized to involve farmed shrimp acquiring and/or selected for an ability to mount elevated antisense ssRNA-based and/or dsRNA-based antiviral responses (Flegel 2007, 2020, 2009).

EVEs comprised of reordered, inverted, repeated, and missing IHHNV genome fragments would be expected to invalidate many PCR tests either designed specifically, or found through use, to amplify IHHNV-EVE dsDNA sequences (Tang *et al.* 2007; Rai *et al.* 2009, 2012; Saksmerprome *et al.* 2011; Cowley *et al.* 2018). As examples, the 356-bp sequence targeted by the 77102F/77353R primer set (Nunan *et al.* 2000) found to amplify both viral ssDNA and EVE dsDNA sequences existed in the S97-EVE and S35-EVE1, but not in the S35-EVE2 sequence. However, nucleotide mismatches at the 3′ terminal position of both primers and at 4 other positions in the 18-mer 77353R primer would likely compromise the capacity of this primer set test to amplify these EVEs. In contrast, neither EVE sequence possessed intact fragments spanning regions amplified by primer sets 392F/R (392 bp) and 389F/R (389 bp) recommended by the World Organisation for Animal Health as useful for amplifying divergent IHHNV strains as well as IHHNV-EVE type A and B sequences, or primer set MG831F/R (831 bp) designed specifically to amplify known linear IHHNV-EVE types (Tang *et al.* 2007). Similarly, the region targeted by a real-time PCR primer set designed to specifically amplify IHHNV-EVE type A sequences was absent from the S97-EVE and S35-EVE1, but present, albeit with some primer mismatches, in the S35-EVE2 sequence (Cowley *et al.* 2018).

Variability among individual *P. monodon* in EVE sequences amplified by a suite of 10 PCR primer sets covering overlapping regions of complete linear IHHNV-EVE sequence have been interpreted to suggest the random integration of IHHNV genome fragments (Saksmerprome *et al.* 2011). While the jumbled fragments in the IHHNV-EVEs described here might explain these, the diversity in EVE makeup suggested by these data would require jumbled EVEs to be characterized in larger numbers of *P. monodon*, or other penaeid species susceptible to IHHNV infection. Such broader information will also be important to devising PCR methods to detect jumbled IHHNV-EVE sequences more reliably.

## Conclusions

Using PacBio long-read data with Illumina short-read polishing together with 10× Genomics and Hi-C scaffolding, this study generated a draft genome assembly and annotation of a black tiger shrimp (*P.* *monodon*) originating from Australia. The assembly represents the first to be produced from this geographically isolated and genetically distinct population (Vu *et al.* 2021). The assembly therefore adds to the genetic resources available for *P. monodon* and Penaeid shrimp in general, and will assist investigations into their evolution and genome expansion resulting from transposable elements. Of the *P. monodon* genome features, the high prevalence of general repeats is the most remarkable, and especially the high content of SSRs even in comparison to other crustacean species. Another unexpected feature was the existence of a previously undescribed IHHNV EVE located between a repeated host sequence. Rather than being comprising of a linear sequence of all or part of the approximately 3.9-kb IHHNV genome, the EVE comprised of a conglomerate of reordered, inverted, and repeated IHHNV genome fragments. Searches of genome assemblies available for *P. monodon* from Thailand, Vietnam, and China indicated with variable confidence, depending on assembly quality, that each contained a similarly jumbled IHHNV-EVE inserted at the same genome location. The fragmented and rearranged nature of these EVEs has implications for detecting them with currently available PCR tests. The presence of multiple inverted sequences including multiple IHHNV RNA transcription promoter elements also has implications for them expressing virus-specific dsRNA capable of interfering with exogenous IHHNV replication. The complexity of the rearranged IHHNV genome fragments comprising the EVEs begs many questions related to how long they have existed in the genomes of genetically diverse *P. monodon*, as well as to what processes have led to their integration at a specific genome location, to the IHHNV genome fragments becoming rearranged and to the apparent multiplication of an RU comprised of highly defined inverted sequences derived from the 5′-terminal region of the IHHNV genome.

## Data availability

Raw and assembled sequence data generated by this study have been deposited in GenBank BioProject PRJNA590309, BioSample SAMN13324362. PacBio and Illumina raw data can be found under accession numbers SRR10713990–SRR10714025. The final scaffolded assembly can be found under accession JAAFYK000000000. RNA-seq data used for annotation originated from an earlier study (Huerlimann *et al.* 2018). The gene models and annotation can be found on Dryad (https://doi.org/10.5061/dryad.f4qrfj6xh).

Supplemental material is available at *G3* online.

## Supplementary Material

jkac034_Supplemental_Material

jkac034_Supplemental_Figures

jkac034_Supplemental_Tables
