# Supplementary material for: Genome assembly of the Australian black tiger shrimp (Penaeus monodon) reveals a novel fragmented IHHNV EVE sequence
Source: G3 (Bethesda). 2022 Feb 10;12(4):jkac034. doi: 10.1093/g3journal/jkac034 (PMC8982415; doi:10.1093/g3journal/jkac034)
Supplement: jkac034_Supplemental_Material [file jkac034_supplemental_material.docx]

# PCR-free library construction (Broad Institute Protocol)

Illumina PCR-free fragment shotgun libraries were prepared using the ‘with-bead pond library’ construction protocol described by Fisher et al. [PMID: 21205303] with the following modifications:

Fragmented DNA was then cleaned up with 0.6x Agencourt AmPure XP SPRI beads and eluted in 40 μl Tris-HCl pH8.0, following manufacturer’s recommendations (Beckman Coulter). DNA fragments were then further cleaned up with 3.0x Agencourt AmPure XP SPRI beads, following manufacturer’s recommendations (Beckman Coulter), but DNA was not eluted from the SPRI beads. Then using the KAPA Library Preparation Kit reagents (KAPA Biosystems, Catalog # KK8241), DNA fragments bound to the SPRI beads were subjected to end repair, A-base tailing and Illumina ‘PCR-free’ TruSeq adapter ligation (Illumina, Catalog FC-121-3001) following manufacturer’s recommendations (KAPA Biosystems). A second 0.7x SPRI clean-up was performed following adapter ligation to remove adapter dimers and library fragments below ~150 bp in size. No library PCR amplification enrichment was performed. Sequence ready Illumina PCR-free library was then eluted off the SPRI beads following manufacturer’s recommendations (Beckman Coulter). Libraries were quantified with quantitative PCR using KAPA Library Quant kit (KAPA Biosystems, Catalog # KK4824) and an Agilent Bioanalyzer High Sensitivity Chip (Agilent Technologies) following the manufacturer’s recommendations.

## Required Consumables:

KAPA Library Preparation Kit - Low throughput, "with bead" (10 rxn) KK8230 (07 137 923 001)

KAPA Library Preparation Kit - Low throughput, "with bead", PCR-free (10 rxn) KK8231

KAPA Library Preparation Kit - Low throughput, "with bead” (50 rxn) KK8232 (07 137 974 001)

KAPA Library Preparation Kit - Low throughput, "with bead", PCR-free (50 rxn) KK8233

KAPA Library Preparation Kit - High throughput, "with bead" KK8234 (07 138 008 001)

KAPA Library Preparation Kit - High throughput, "with bead", PCR-free KK8235

AMPure XP, 450 ml A63882

## Fragment DNA

1. Normalize gDNA samples to 10 ng/μl in Buffer EB
2. Transfer 52.5 μl to a new Covaris tubes
3. Tubes can be held upright in s 0.3 ml PCR plate
4. Briefly centrifuge Covaris tube (600 x 6, 5 s)
5. Fragment DNA with the following settings

### Table 1: Covaris E220 Settings

| Parameter | Setting |
| --- | --- |
| Target BP (Peak) | 400 |
| Peak Incident Power | 175 |
| Duty cycle | 5% |
| cycles per burst | 200 |
| Treatment time | 45 seconds |
| Temperature | 7°C |
| Water Level | 6 |
| Sample Volume | 50 μl |
| E220 - Intensifier (pn500141) | YES |

## 1^st^ Clean-up of fragmented DNA.

*Ensure AMPure XP beads have equilibrated to RT for at least 30 minutes*

1. Transfer **50 μl** of sheared DNA from each Covaris tube to a new well of a 0.3 ml PCR plate.
2. Vortex AMPure XP beads for 1 minute to disperse beads.
3. Add **30 μl (0.6X volume)** of AMPure XP beads to each well containing fragmented DNA. Mix well by pipetting the entire volume up and down **10 times**
4. Incubate plate at RT for **5 minutes**
5. Place plate on magnetic stand and stand at RT until bead have collected to sides of the wells (5 minutes)
6. Remove and discard the supernatant (80 μl)
7. Add 200 μl of freshly prepared 80% EtOH to each well (without disturbing the beads)
8. Incubate at RT for 30 second, then remove and discard the supernatant.
9. Repeat steps 7 and 8 for a total of two washes.
10. Leave plate on the magnetic stand at RT for **5 minutes**. Remove any residual EtOH with a 10 μl pipette**.**
11. Add 40 μl of Buffer EB to each well. Remove plate from the magnetic stand and resuspend the beads by gently dispensing the buffer over the bead until fully resuspended. Gently mix by pipetting the entire volume up and down 10 times.
12. Incubate plate at RT for **2 minutes**
13. Place plate on magnetic stand and stand at RT until bead have collected to sides of the wells (5 minutes)
14. Transfer supernatant to a new 0.3 ml PCR plate.

## 2^nd^ Clean-up of fragmented DNA

1. Vortex AMPure XP beads for 1 minute to disperse beads.
2. Add **120 μl** (3.0X volume) of AMPure beads to each well to each well containing fragmented DNA. Mix well by pipetting the entire volume up and down 10 times
3. Incubate plate at RT for 5 minutes
4. Place plate on magnetic stand and stand at RT until bead have collected to sides of the wells (5 minutes)
5. Remove and discard the supernatant (80 μl)
6. Add 200 μl of freshly prepared 80% EtOH to each well (without disturbing the beads)
7. Incubate at RT for 30 second, then remove and discard the supernatant.
8. Repeat steps 7 and 8 for a total of two washes.
9. Leave plate on the magnetic stand at RT for 5 minutes. Remove any residual EtOH with a 10 μl pipette**.**
10. Add 50 μl of Buffer EB to each well. Remove plate from the magnetic stand and resuspend the bead by gently dispensing the buffer over the bead until fully resuspended. Gently mix pipetting the entire volume up and down 10 times.
11. Proceed directly to End Repair- DO NOT SEPARATE THE BEADS

## End Repair of Fragmented DNA

1. Prepare End Repair Master Mix (table 2) and store on ice until required.

### Table 2: End-repair master mix

| Component | 1 Library | 8 Libraries (+5%) |
| --- | --- | --- |
| Water | 8 μl | 97 μl |
| 10X KAPA Repair Buffer | 7 μl | 59 μl |
| KAPA End Repair Enzyme Mix | 5 μl | 42 μl |
| Total Volume | 20 μl | 168 μl |

1. Add **20 μl** of End Repair Master Mix to each well
2. Mix thoroughly by pipetting the entire volume up and down 10 times
3. Incubate at **20°C for 30 minutes**

## A-Tailing of Fragmented DNA

1. After incubation, briefly spin down reaction
2. To each well/tube add **120 μl** of PEG/NaCl SPRI solution (1.7X)
3. Mix thoroughly by pipetting the entire volume up and down 10 times
4. Incubate plate at RT for 15 minutes (to allow DNA to bind to bead)
5. While incubating, prepare **A-Tailing Master Mix** (table 3) and store on ice until required.

### Table 3: A-Tailing Master Mix

| Component | 1 Library | 8 Libraries (+5%) |
| --- | --- | --- |
| Water | 42 μl | 97 μl |
| 10X KAPA A-Tailing Buffer | 5 μl | 42 μl |
| KAPA A-Tailing Enzyme | 3 μl | 25 μl |
| Total Volume | 50 μl | 420 μl |

1. Place plate on magnetic stand and stand at RT until bead have collected to sides of the wells (5 minutes)
2. Remove and discard the supernatant (190 μl)
3. Add 200 μl of freshly prepared 80% EtOH to each well (without disturbing the beads)
4. Incubate at RT for 30 second, then remove and discard the supernatant.
5. Repeat steps 7 and 8 for a total of two washes.
6. Leave plate on the magnetic stand at RT for 5 minutes. Remove any residual EtOH with a 10 μl pipette.
7. Add **50 μl** of A-Tailing Master Mix to each well/tube. Remove plate from the magnetic stand and resuspend the bead by gently dispensing the reaction mix over the beads until fully resuspended. Gently mix pipetting the entire volume up and down 10 times.
8. Incubate the A-tailing reaction at **30°C for 30 minutes**

## A-tailing clean up

1. After incubation, briefly spin down reactions
2. To each well/tube add **90 μl** of PEG/NaCl SPRI solution **(1.8X)**
3. Mix thoroughly by pipetting the entire volume up and down 10 times
4. Incubate plate at RT for 15 minutes (to allow DNA to bind to bead)
5. While incubating, prepare **Ligation Master Mix** (table 4) and store on ice until required.

### Table 4: Ligation Master Mix

| Component | 1 Library | 8 Libraries (+5%) |
| --- | --- | --- |
| Water | 30 μl | 252 μl |
| 5 X KAPA Ligation Buffer | 10 μl | 84 μl |
| KAPA T4 DNA Ligase | 5 μl | 42 μl |
| Total Volume | 45 μl | 387 μl |

1. Place plate on magnetic stand and stand at RT until bead have collected to sides of the wells (5 minutes)
2. Remove and discard the supernatant (80 μl)
3. Add 200 μl of freshly prepared 80% EtOH to each well (without disturbing the beads)
4. Incubate at RT for 30 second, then remove and discard the supernatant.
5. Repeat steps 7 and 8 for a total of two washes.
6. Leave plate on the magnetic stand at RT for 5 minutes. Remove any residual EtOH with a 10 μl pipette.
7. To each well add the following
   1. **45 μl** of Ligation Master Mix
   2. **5 μl** of TruSeq DNA Adapter
8. Resuspend the bead by gently dispensing the reaction mix over the the bead until fully resuspended. Gently mix pipetting the entire volume up and down 10 times.
9. Incubate the A-tailing reaction at **20°C for 15 minutes**

## Clean-up of Ligation reaction

1. After incubation, briefly spin down reaction
2. To each well/tube add **50 μl** of PEG/NaCl SPRI solution (1X)
3. Mix thoroughly by pipetting the entire volume up and down 10 times
4. Incubate plate at RT for 15 minutes (to allow DNA to bind to bead)
5. Place plate on magnetic stand and stand at RT until bead have collected to sides of the wells (5 minutes)
6. Remove and discard the supernatant (150 μl)
7. Add 200 μl of freshly prepared 80% EtOH to each well (without disturbing the beads)
8. Incubate at RT for 30 second, then remove and discard the supernatant.
9. Repeat steps 7 and 8 for a total of two washes.
10. Leave plate on the magnetic stand at RT for 5 minutes. Remove any residual EtOH with a 10 μl pipette.
11. Add **100 μl** of Buffer EB to each well. Remove plate from the magnetic stand and resuspend the bead by gently dispensing the buffer over the bead until fully resuspended. Gently mix pipetting the entire volume up and down 10 times.
12. Incubate plate at RT for 2 minutes
13. Place plate on magnetic stand and stand at RT until bead have collected to sides of the wells (5 minutes)
14. Transfer supernatant to a new 0.3 ml PCR plate
15. To each well/tube add **70 μl** of well mixed AMPure XP beads (0.7X)
16. Mix thoroughly by pipetting the entire volume up and down 10 times
17. Incubate plate at RT for 15 minutes (to allow DNA to bind to bead)
18. Place plate on magnetic stand and stand at RT until bead have collected to sides of the wells (5 minutes)
19. Remove and discard the supernatant (1700 μl)
20. Add 200 μl of freshly prepared 80% EtOH to each well (without disturbing the beads)
21. Incubate at RT for 30 second, then remove and discard the supernatant.
22. Repeat steps 7 and 8 for a total of two washes.
23. Leave plate on the magnetic stand at RT for 5 minutes. Remove any residual EtOH with a 10 μl pipette.
24. Add **22.5 μl** of re-suspension buffer (Buffer EB) to each well. Remove plate from the magnetic stand and resuspend the bead by gently dispensing the buffer over the bead until fully resuspended. Gently mix pipetting the entire volume up and down 10 times.
25. Incubate plate at RT for 2 minutes
26. Place plate on magnetic stand and stand at RT until bead have collected to sides of the wells (5 minutes)
27. Transfer 20 μl of supernatant to new 0.3 ml PCR plate or 1.5 ml tube
