# Supplementary material for: Genome assembly of the Australian black tiger shrimp (Penaeus monodon) reveals a novel fragmented IHHNV EVE sequence
Source: G3 (Bethesda). 2022 Feb 10;12(4):jkac034. doi: 10.1093/g3journal/jkac034 (PMC8982415; doi:10.1093/g3journal/jkac034)
Supplement: jkac034_Supplemental_Figures [file jkac034_supplemental_figures.docx]

**Supplementary Figure 1** Sequences extending toward the 5’-terminus of IHHNV and IHHNV-EVE sequences extending from 18 bp inverted repeats flanking the outside boundaries of the RU1 and RU6 components of the six RU block as well as associated with three partial RU sequences (pRUa, pRUb, pRUc) positioned just upstream of RU1 in the S97-EVE

....GAGTGAAGAGGCTATTCCAAGTGACTAAGGACAATTTTGGAACATGGAAGATACGAATAACCATCCATGGCAGTCAATACCTTCGCAGGAAACCGTTACAACCTATGAC**|**GTCATAGGTCCTATATAAGAG **5’-pRUa-RC**

.....................................CTATAATTATCCATGCTAAGATTTCTGGTACCACGGATGTCATTGTTGTTCGCAGGAAACCGTTACAACCTATGAT**|**GTCATAGGTCCTATATAAGAG **3’-pRUa-RC**

....TTGCTGACAAAAAACTTGATAAAATTAACACTCTAGTTCTACAAGGACCGACAGGAACAGGCAAATCTCTGACCATTGGCATTCGCAGGAAACCGTTACAACCTATGAC**|**GTCATAGGTCCTATATAAGAG **5’-pRUb**

...................................AGGTATTTCGTCATTATGAGATTATTGTCTCACCTTTCCTGCGAAGC**|**GCTCCGC-GGAAACCGTTACAACCTATGAC**|**GTCATAGGTCCTATATAAGAG **5’-pRUc**

....ACTACCGAACAACTACTTAATATGTCTGAAGAATTGTTCAAGTTTTCAGACGAGGAAGACAATGGTTTC-TGCGGAGC**|**GCTTCGCAGGAAACCGTTACAACCTATGAC**|**GTCATAGGTCCTATATAAGAG **5’-RU1**

**.........................................**GCGCAAAGACCGTTAACAATTTATGTGACGTCATCACGCCAGTTTCCGCAGGAAACCGTTACAACCTATGAC**|**GTCATAGGTCCTATATAAGAG **3’-RU6-RC**

5’-TAGAGCGCGAAGCGCGAGTATCCATCATTTAAATTAGTGGTATGACGTCACATATTAAGTTAACGGTTTC-TGCGAAGC**|**GCTTCGCAGGAAACCGTTACAACCTATGAC**|**GTCATAGGTCCTATATAAGAG **AF218266.2**

5’-GAGGCTATTCCAAGTGACTAAGGACAATTTTGGAACATGGAAGATACGAATAACCATCCATGGCAGTCAATACCTTCGCAGGAAACCGTTACAACCTATGAC**|**GTCATAGGTCCTATATAAGAG **EU675312.1**

5’-TCATGAAGCGCGAGTATCCATCATTTAAATTAGTGGTATGACGTCACATATTAAGTTAACGGTTTC-TGCGAAGC**|**GCTTCGCA-GAAACCGTTACAACCTATGAC**|**GTCATAGGTCCTATATAAGAG **KF907320.1**

5’-TCATAGGTTGTAACGGTTTCCTGCG-AGC**|**GCTTCGCA-GAAACCGTTACAACCTATGAC**|**GTCATAGGTCCTATATAAGAG **AF273215.1**

Repeat unit (RU); Partial RU (pRU); reverse compliment sequence (RC); nucleotide not shown (.); conserved 18 bp inverted repeat nucleotides (yellow shading) and inversion point (**|**);conserved 39-40 bp inverted repeat nucleotides (green shading) and inversion point (**|**); space added to maintain alignment (-); P2 transcriptional promoter TATA-box; 5’-terminal IHHNV-EVE nucleotide sequence (EU675312.1) conserved with 5’-pRUa-RC sequence beyond the conserved 20 bp portion of the 39-40 bp inverted repeat (cyan shading)

**Supplementary Figure 2** Sequences in the four RU block of the Thai S35-EVE1 upstream of RU1, downstream of RU4 and at the internal RU1-RU2, RU2-RU3 and RU3-RU4 boundaries

5’-..TTGTCTCACCTTTCCTGCGAAGC**|**GCTCCGCAGAAACCGTTACAACCTATGAC**|**GTCATAGGTCCTATATAAGA**[RU1]**..-3’ **5’-RU1**

5’-..**[RU4]**TCTTATATAGGACCTATGAC**|**GTCATAGGTTGTAACGGTTTCCTGCGGAAACT..-3’ **3’-RU4**

5’-..ACCTATGAC**|**GTCATAGGTTGTAACGGTTTCCTGCGAAGC**|**GCTCCGCAGAAACCGTTACAACCTATGAC**|**GTCATAGGT..-3’ **RU1-RU2, RU2-RU3, RU3-RU4 boundaries**

Repeat unit (RU); nucleotide not shown (.); conserved 18 bp inverted repeat nucleotides (yellow shading) and inversion point (**|**); conserved 41 bp inverted repeat nucleotides (green shading) and inversion point (|); P2 transcriptional promoter TATA-box
